# Impact of COVID-19 lockdown on suicide attempts: A retrospective analysis of the springtime admissions to the trauma resuscitation room at the Medical University of Vienna from 2015–2020

**DOI:** 10.1007/s00508-021-01839-6

**Published:** 2021-03-31

**Authors:** Greta L. Carlin, Josef S. Baumgartner, Timon Moftakhar, Daniel König, Lukas L. Negrin

**Affiliations:** 1 Department of Orthopaedics and Trauma-Surgery, Medical University of Vienna, Vienna, Austria; 2 Clinical Division of Social Psychiatry, Department of Psychiatry and Psychotherapy, Medical University of Vienna, Vienna, Austria

**Keywords:** COVID-19 pandemic, Suicide attempts during lockdown, Coronavirus lockdown, Polytrauma, Suicide prevention

## Abstract

**Background:**

In response to the current coronavirus disease 2019 (COVID-19) pandemic the Austrian government issued a lockdown from 16 March to 15 May 2020. As periods of economic and emotional burden have proven to detrimentally affect people’s psychological health, healthcare officials warned that the strict measures could have a serious impact on psychological health, leading to an increase in suicide attempts. Thus, the objective was to provide evidence for this assumption.

**Methods:**

All adult patients admitted to the trauma resuscitation room of the Medical University of Vienna during the lockdown period following a suicide attempt were included in this analysis, forming the study group. Suicidal patients treated during the same period in 2015, 2016, 2018, and 2019 were pooled to create the control group. The year 2017 was excluded because another major hospital in Vienna was partially closed due to a change in infrastructure, resulting in an increased number of severely injured patients treated at our department. As the lockdown caused a markedly decreased number of patients admitted due to other reasons than suicide the actual number was replaced with the average number of patients admitted in the relevant previous 4 years.

**Results:**

By comparing the study and the control group based on this realistic estimate we revealed an increase in attempted suicides during the lockdown period (*p* = 0.001). Demographic, mental health-related, and injury specific patient data did not differ between the groups.

**Conclusion:**

The results strongly urge for an improvement in crisis intervention and suicide prevention measures in the event of a future lockdown.

## Introduction

Suicide is the second leading cause of traumatic death worldwide, surpassed only by road traffic collisions [1]. The World Health Organization (WHO) estimated that in 2016 around 793,000 people died by suicide and while the global total number seems to be decreasing, various countries such as the USA, South Korea and Japan are reporting rising trends [2–6]. In 2018, 1209 people died from suicide in Austria, the most common methods were hanging (45%), shooting (19%), jumping from a height and poisoning (10% each), jumping in front of a moving object (7%), drowning (3%), cutting, burning or causing an accident [7].

Suicide attempts are a serious, potentially preventable healthcare problem. On the one hand, trying to commit suicide may be a cry for help, a conscious or unconscious method for getting others to recognize just how badly the individual is feeling. On the other hand, contemplating suicide may be caused by a very pronounced death wish. Considering the mechanisms of injury and the physics behind them, it seems clear that the first group of individuals attempting suicide preferably choose methods, such as cutting, which mostly injure only one body region (monotrauma), not immediately leading to death and allowing the injured to call for help in case they change their mind; however, individuals, who are determined to commit suicide, preferably select irrevocable methods that result in multiple injuries (polytrauma), unlikely to survive, such as jumping from great heights or jumping in front of a train. Moreover, polytrauma patients pose a unique challenge to the team treating them, frequently requiring a multidisciplinary approach with the individual outcome remaining hard to predict [8], resulting in a significant socioeconomic burden. By investigating the changes in the number of patients admitted to the trauma resuscitation room of the Medical University of Vienna following intentionally caused trauma (differentiating between polytrauma and nonpolytrauma) we were able to evince the effect of the lockdown on people’s psychological health.

The current COVID-19 pandemic has spread rapidly around the globe and become an international health concern [9]. Furthermore, it also negatively impacts the economic stability of various countries and thus directly affects the financial livelihood of many people [10]. In order to flatten the hospitalization curve and thus not overload the healthcare system, the Austrian government issued a set of strict measures and social restrictions beginning on 16 March 2020. All non-essential businesses were closed, and most workplaces established home office working. Kindergartens, schools and universities were shut for the foreseeable future. Especially senior citizens were asked to remain at home given their increased vulnerability [11]. These measures lasted up to 15 May 2020, when the lockdown officially ended. Similar measures were introduced in many other countries. Accordingly, the possibility to get in touch with medical caregivers was reduced as ambulatory healthcare for non-emergency patients could not be provided as extensively as usual. Additionally, individual patient evaluation as well as various treatment options, such as elective surgery were drastically affected by COVID-19 [12–17]. Furthermore, by social distancing from friends and family, individuals appeared to be completely left alone with often compromising afflictions, hence causing alarming psychological distress [18].

From the start, healthcare providers worldwide warned that the lockdown for the current COVID-19 pandemic could result in unemployment and lead to increased anxiety and depression [10, 19, 20]. In addition, social distancing measures could confluence to increase known risk factors for suicidal behavior, such as living alone, loneliness and social isolation [21, 22]. The possibility of an increasing occurrence of suicide attempts, especially in more vulnerable populations, such as senior citizens and those already suffering from mental illness was further discussed [23–26]. Until now there are no data on the effects of the COVID-19 crisis on suicide attempts.

The aim of the present study was to analyze how many of the patients admitted to the trauma resuscitation room of the level 1 Trauma Centre of the Medical University of Vienna during the COVID-19 lockdown in Austria were injured due to a suicide attempt by intentionally caused trauma. Secondly, we investigated how many of those were classified as a polytrauma and whether these numbers or any of the demographic, socioeconomic and health-specific variables collected differed from the patients admitted following a suicide attempt during the same time period in the previous 5 years. The authors hoped to thereby gain more insight for healthcare professionals should a future lockdown or pandemic occur.

## Methods

### Data source

Our retrospective data analysis, which was approved by the local ethics committee (EK no: 1607/2020), focused on all patients (1) with a minimum age of 18 years, (2) who were admitted to the level 1 Trauma Centre of the Medical University of Vienna during the lockdown period established by the Austrian government (16 March–15 May 2020), and (3) for whom treatment in the trauma resuscitation room (shock room) was considered appropriate due to the injury mechanism. These patients were subdivided into trauma victims and individuals who had attempted suicide. Of the latter, demographic data including age, gender, outcome (survival/fatality), ethnic minority background, known substance abuse, known psychiatric diagnosis, previous suicide attempt, previous psychiatric treatment, abbreviated injury scale (AIS) for the regions head or neck (AIS_Head_), face (AIS_Face_), thorax (AIS_Thorax_), abdominal or pelvic contents (AIS_Abdomen_), extremities or pelvic girdle (AIS_Extremities_), and external (AIS_External_), as well as injury severity score (ISS) and zip code of residence district were extracted from patients’ medical charts. Of our study group, a special focus was set on polytrauma victims, defined by an ISS ≥ 16. In order to be able to form a valid control group all patients who were admitted to the level 1 Trauma Centre of the Medical University of Vienna following a suicide attempt from 16 March to 15 May in the years 2015–2019, were assessed. In general, survival referred to the period of hospitalization.

### Statistics

All patient records were pseudoanonymized and deidentified prior to statistical analysis that was performed using the SPSS system (IBM, Armonk, NY, USA, version 26). Normally distributed variables are presented as mean ± standard deviation, non-normally distributed variables and categorical data are characterized by median and range in square brackets, and dichotomous characteristics are displayed by absolute and relative frequencies. The Mann-Whitney U-test was applied to compare continuous variables, whereas categorical data were analysed by means of the χ^2^-test. In general, a *p*-value < 0.05 was considered significant.

## Results

**Fig. 1:**
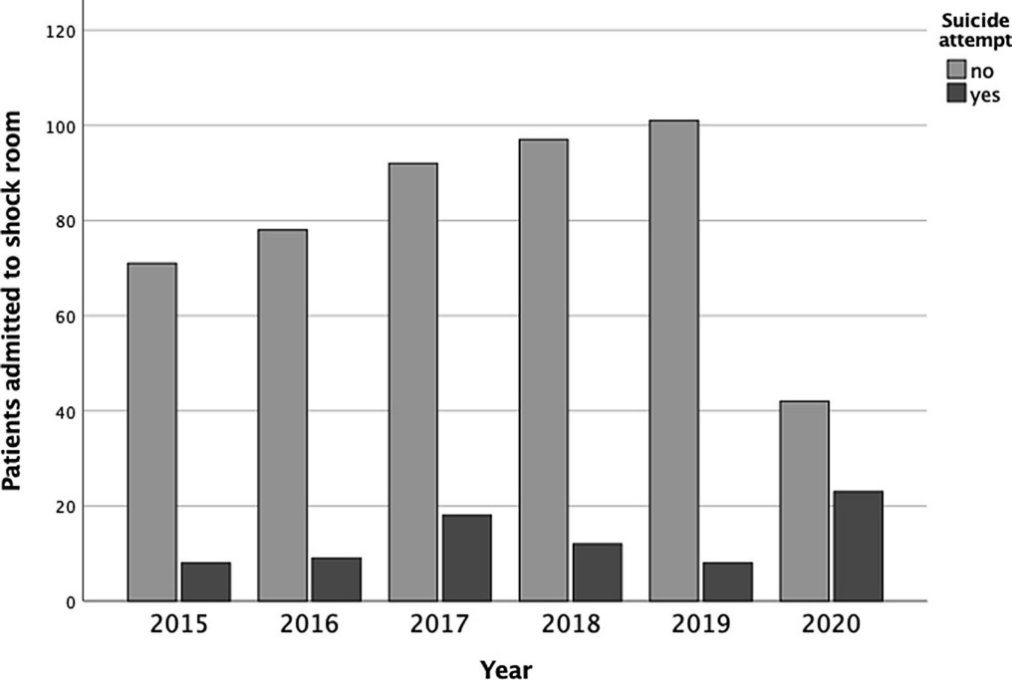
Patients admitted to resuscitation room (suicidal vs. non-suicidal) in the same 2‑month period from 2015 to 2020

**Fig. 2:**
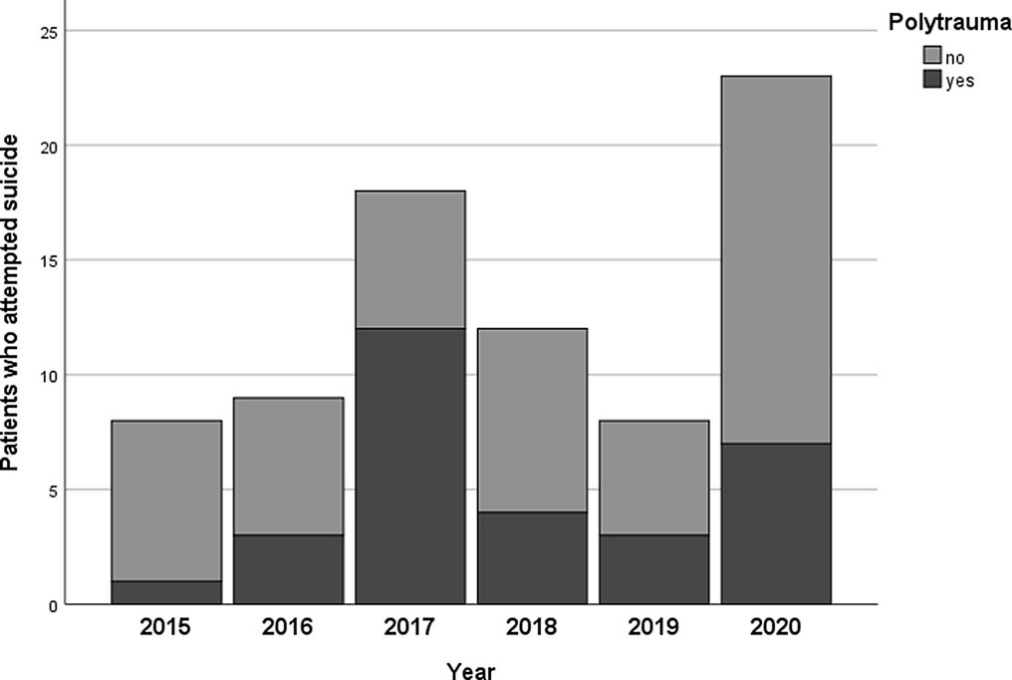
Patients after suicide attempt (polytrauma vs. non-polytrauma) in the same 2‑month period from 2015 to 2020

Our database search provided the numbers presented in Table 1. Fig. 1 displays the absolute frequencies of accidental and intentionally caused trauma depending on the year of admission, revealing a different distribution (*p* < 0.001). Fig. 2 shows the proportion of polytrauma victims based on all patients who had attempted suicide, which did not differ significantly across the years 2015–2020 (*p* = 0.102).

**Table 1:** Number of patients admitted to the trauma resuscitation room according to years

Number	Year					
	2015	2016	2017	2018	2019	2020
Admissions	79	87	110	109	109	65
Attempted suicides	8 (10.1%)	9 (10.3%)	18 (16.4%)	12 (11.0%)	8 (7.3%)	23 (35.4%)
Polytrauma	1 (1.3%)	3 (3.4%)	12 (10.9%)	4 (3.7%)	3 (2.8%)	7 (10.8%)

Since we detected a spike of suicide-associated polytrauma patients admitted to our institution for the 2‑month period in 2017 we further investigated the reason for this occurrence, revealing that data were not comparable (as specified in the discussion) and thus had to be excluded from our statistical analysis. In consequence, our control group was formed by all patients admitted to the trauma resuscitation room in 2015, 2016, 2018, and 2019 following a suicide attempt, whereas all suicidal patients treated in 2020 were combined to our study group. As an effect of the lockdown the number of patients admitted due to other causes than suicide decreased markedly in 2020, necessitating a realistic estimate of this number to shed light on the research question regarding a potential increase in suicides as hypothesized. We decided to replace the actual number of patients admitted for other reasons than suicide in 2020 with 87, the average number of patients admitted in 2015, 2016, 2018, and 2019, resulting in a total of 110 fictive admissions to the trauma resuscitation room in 2020, including 20.9% attempted suicides and 6.4% suicidal polytraumas.

Based on this estimate we revealed a significantly higher proportion of attempted suicides in all patients admitted to the trauma resuscitation room (*p* = 0.001) during the lockdown period in 2020 compared to the same 2‑month time period in the pooled previous 4 years, whereas no difference in the proportion of suicidal polytrauma could be found (*p* = 0.084).

In order to detect any differences in demographic, injury-specific and mental health-related data, we compared the patients in the study group with those in the control group. The results are presented in Table 2. As expected, subgroup analyses did not reveal significant differences due to the small number of patients (*p* ≥ 0.210). Moreover, significant differences in injury severity, assessed by the AIS, could not be calculated (*p* ≥ 0.086).

**Table 2:** Demographic, injury-specific and mental health-related data of the patients in the control and in the study group

	Control group	Study group
*Number of patients*	37	23
*Deceased*	2 (5.4%)	1 (4.3%)
*Age (years)*	43.2 ± 17.6	38.7 ± 12.2
*Male:female*	29:8	17:6
*ISS*	4 [1–75]	4 [1–43]
*Polytrauma*	11 (29.7%)	7 (30.4%)
*Known substance abuse*	18 (48.6%)	15 (65.2%)
*Known psychiatric disease*	32 (86.5%)	19 (82.6%)
*Previous suicide attempt*	13 (35.1%)	9 (39.1%)
*Previous psychiatric treatment*	25 (67.6%)	12 (52.2%)
*Minority background*	10 (27.0%)	9 (39.1%)
**Suicide method used**		
Jump from a height	9 (24.3%)	10 (43.5%)
Jump in front of a moving object	5 (13.5%)	2 (8.7%)
Cutting	16 (43.2%)	6 (26.1%)
Driving off the street	3 (8.1%)	1 (4.3%)
Self-immolation	0 (0%)	1 (4.3%)
Hanging	1 (2.7%)	2 (8.7%)
Shooting	1 (2.7%)	0 (0%)
Self-castration	1 (2.7%)	0 (0%)
Ingestion of poison, harmful substance etc.	1 (2.7%)	1 (4.3%)

*ISS* Injury Severity Score

Of the 23 patients in our study group, 7 sustained polytrauma (age 35.1 ± 13.5 years; ISS, 41 [22–43]), 5 males jumped from a height, 1 female jumped in front of a train (she was the only patient who died) and 1 female performed self-immolation. The control group included 11 individuals suffering from polytrauma (age 34.9 ± 17.7 years; ISS, 29 [14–75]). The mechanisms of injury were jumping from a height (4), jumping in front of a moving object (4), cutting (1) and driving off the street (2).

**Fig. 3:**
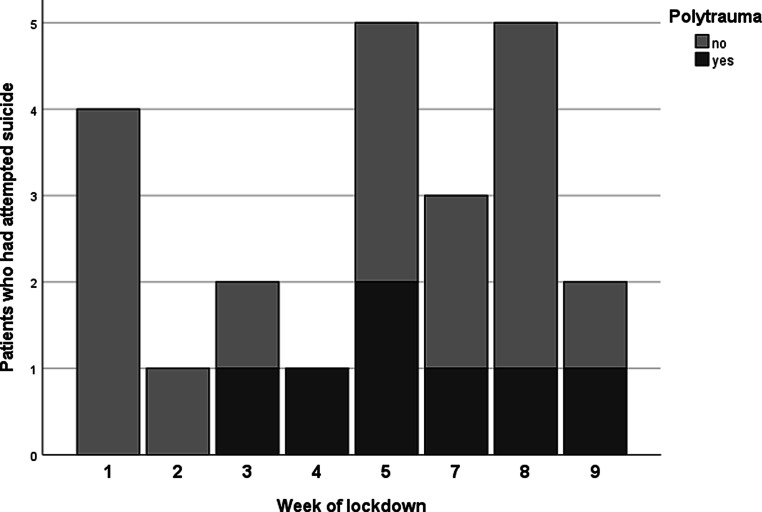
Patients after suicide attempt (polytrauma vs. non-polytrauma) in each week of the lockdown

In the study group the subdivision of the patients in those sustaining/not sustaining polytrauma according to the week of admission during lockdown is presented in Fig. 3, not reaching statistical significance (*p* = 0.577).

Finally, no cluster regarding shared areas of residence could be detected for individuals admitted due to suicide attempts.

## Discussion

### Main findings

During the national COVID-19 lockdown in Austria we identified a higher percentage of suicide-associated admissions to the resuscitation room of our level 1 Trauma Centre compared to the same 2‑month time period in each of the previous 5 years. These differences did not necessarily indicate that there were more suicide attempts in 2020 as the number of patients admitted due to other reasons than suicide was lower than average. Moreover, it turned out that the year 2017 was not comparable to the other years 2015–2019, necessitating exclusion of the data for 2017 from our analysis. Since the number of patients admitted to the trauma resuscitation room due to reasons other than suicide markedly decreased during the lockdown, we replaced this number by the mean number of patients admitted in 2015, 2016, 2018, and 2019 due to reasons other than suicide. Even after this adaptation a higher percentage of attempted suicides (*p* = 0.001) was revealed in the study group compared to the control group; however, a higher percentage of patients suffering an intentionally caused polytrauma could not be revealed (*p* = 0.084).

Regarding the mechanism of injury, most attempted suicides were performed by jumping from a height during the COVID-19 lockdown, whereas the majority of patients in the control group had presented after trying to kill themselves through cutting. Finally, the ratio of males and females, age and overall injury severity as well as known risk factors for suicide, such as ethnic minority status, socioeconomic background, substance abuse, pre-existing psychiatric diagnosis, previous suicide attempt and previous psychiatric treatment, did not differ significantly between the study and the control group.

### Interpretation of the findings

As the lockdown measures took effect and people were advised to “stay at home” we noticed a reduced number of admittances to the resuscitation room of our level 1 trauma centre following a traumatic injury, which can be explained by the reduced number of accidents generally occurring at the workplace, in traffic or at outdoor activities. While the number of patients admitted to our institution due to accidental traumatic injury decreased, the number of patients admitted following an intentional traumatic injury due to a suicide attempt increased. Therefore, the percentage of patients admitted following a suicide attempt during the COVID-19 lockdown was significantly higher compared to the previous 5 years.

The reasons that lead an individual to suicidal behavior are still not fully understood, as the causes are complex and multifactorial [27]. One of the reasons for the significantly higher percentage of suicide-associated admissions to our resuscitation room during the COVID-19 lockdown might be the increased prevalence of depression and anxiety symptoms in this period, compared to previous epidemiological data in Austria [28]. Interestingly, when Pieh et al. performed the same survey they conducted in Austria on a study population in the United Kingdom, they found that the prevalence of severe depressive, anxiety and insomnia symptoms was about three times higher there than in Austria [29]. These findings lead to the hypothesis that other countries might have experienced an even higher increase in the rate of suicide attempts than our Austrian study population.

A level 1 trauma centre is a verified facility that provides the highest level of surgical care to trauma patients and has a full range of specialists and equipment available 24 h per day. The Medical University of Vienna is the only level 1 Trauma Centre in the greater Vienna region with two resuscitation rooms, where patients can be treated simultaneously. Additionally, it is known that polytrauma patients have a significantly lower mortality when care is obtained at a trauma center [8]. Therefore, it stands to reason that our institution received an influx of major trauma and polytrauma patients during this time period.

The absolute number of suicide-related polytraumas as well as the proportion of suicide-related polytraumas in comparison to all admissions was higher in our study group than in the groups referring to 2015, 2016, 2018, and 2019. Surprisingly, we detected a spike of suicide-associated polytrauma patients admitted to our institution for the 2‑month period in 2017 and further investigated the reasons for this occurrence. During the spring of 2017 another major hospital in Vienna had partially closed its trauma center due to a change in infrastructure [30]. For this reason, data on polytrauma from 2017 are not comparable and were excluded from our analysis. We further studied if a difference in suicide-associated polytrauma admittance could be detected according to the week of admission. Although no suicide-associated polytrauma was treated during the first 2 weeks of the lockdown, no significant difference could be detected; however, this might also be a result of our small sample size. Moreover, we could not find a difference in the mechanism of injury between the study and control groups.

Risk factors associated with higher suicide rates, such as age, gender, substance misuse and mental health concerns, such as depression have been identified [21] and thus at-risk populations defined. It was hypothesized that the number of suicide attempts in the senior population could increase as a result of social isolation during lockdown [26]; however, our data showed no difference in the age distribution of patients who attempted suicide during the lockdown from those in the previous 4 years. Regarding gender, we detected that more males than females attempted suicide in both study and control groups, which is in line with previously published literature [7].

Due to the small number of patients in our study group we could not observe an increase in suicidal behavior isolated for individuals with previous mental health problems, but more generally an increase in suicidality across different groups at risk. Nevertheless, people suffering from psychiatric disorders or mental health conditions tend to be more susceptible to stress than the general population and could therefore experience relapses, new manifestations or substantial worsening of symptoms, such as depression, anxiety, and posttraumatic stress. It follows that all these factors could lead to increased psychological distress and thus lower the inhibition to perform a suicide attempt. Furthermore, many outpatient clinics were closed during the lockdown period and thus regular visits could not take place and the continuance of prescriptions was not always guaranteed, increasing the burden on patients already suffering from mental health symptoms even further [31].

The COVID-19 pandemic has become an amplifier of already existing socioeconomic vulnerabilities and its economic fallout might also lead to further healthcare threats. It is known that unemployed individuals have a higher suicide risk than their employed counterparts and that during spikes of unemployment rates a spike in the number of suicides can also be detected [32–34]. Considering the current global unemployment rates and their projected rise, an increase in suicide attempts should also be expected and prepared for by the mental health community [35, 36]. Unfortunately, we were unable to assess the current employment status of our study population and can therefore not comment if the economic instability led to the increase of suicidality in our study group. We did analyze the various zip codes to determine the socioeconomic background through the patients’ district of residence; however, no geographical cluster could be detected. We also found no association between suicide attempts during the COVID-19 lockdown and belonging to an ethnic minority, even if it has been hypothesized that ethnic minorities, especially migrant populations, such as refugees, migrant workers and asylum seekers could be exponentially more affected by the current pandemic as they are especially vulnerable to the aforementioned dynamics [37].

### Strengths and limitations

Many researchers discussed the possible impact of the COVID-19 lockdown on mental health and suicide attempts; however, to our knowledge this is the first study statistically confirming this hypothesis. By observing the number of suicide-associated admissions to the resuscitation room of our level 1 trauma centre, we are one of the few studies publishing actual data on how many suicide attempts were hospitalized during the lockdown period.

We are aware of the limitations of our study. By the design of our study we had only access to the number and mechanism of injury admitted to the resuscitation room of the level 1 Trauma Centre of the Medical University of Vienna and cannot estimate how many patients received care in other departments or hospitals in the greater Vienna area or Austria in general; however, the absolute number of suicidal acts performed will always be impossible to determine as many individuals do not seek treatment in a hospital or help from mental health professionals after attempting suicide.

## Conclusion

Our findings show that during the lockdown in Austria a significantly higher percentage of patients admitted to the resuscitation room of the level 1 Trauma Centre sustained their injuries due to a suicide attempt by intentional trauma compared to the same 2‑month period in the pooled previous 4 years. Therefore, it is crucial to consider mental health and psychological well-being when establishing lockdown policies. Furthermore, healthcare professionals working in trauma centres and first responders should be aware of the needs of patients admitted after attempting suicide during a lockdown. Therefore, consultation of psychiatric and psychological services should be considered from an early point of intervention. We recommend an early interdisciplinary approach.
